# Acute Heat Stress Alters the Expression of Orexin System in Quail Muscle

**DOI:** 10.3389/fphys.2017.01079

**Published:** 2017-12-19

**Authors:** Phuong H. Nguyen, Elisabeth Greene, Byung-Whi Kong, Walter Bottje, Nicholas Anthony, Sami Dridi

**Affiliations:** Center of Excellence for Poultry Science, University of Arkansas, Fayetteville, AR, United States

**Keywords:** heat stress, oxidative stress, orexin, HSP70, muscle, quail

## Abstract

Accumulating evidences indicate that the hypothalamic neuropeptide orexins or hypocretins are involved in stress-induced responses in mammals. Recently, we found that orexin is expressed and secreted from avian muscle cells, however its regulation is still unknown. In this study, we investigated the effect of heat and oxidative stress, the most challenging stressors in poultry production, on the expression of orexin system in quail muscle tissues and myoblast cell lines. Four week-old genetically selected susceptible and resistant Japanese quail (*Coturnix coturnix Japonica*) lines were exposed to acute heat stress (HS, 37°C for 1.5 h) or maintained at thermoneutral conditions (24°C). Quail myoblast (QM7) cell line was exposed to heat stress (45°C) for 0.5, 1, 2, or 4 h. The control cells were maintained at 37°C. The cells were also treated with several doses of hydrogen peroxide (H_2_O_2_, 10–200 μM) or 4-Hydroxynonenal (4-HNE, 10–30 μM) as oxidative stress. Untreated cells were used as controls. Acute HS significantly induced the expression of HSP70 and down-regulated orexin system in both quail muscle tissue and QM7 cells. Similarly, H_2_O_2_ but not 4-HNE treatment significantly increased HSP70 protein levels and dysregulated the expression of orexin and its related receptors in a dose-dependent manner in QM7 cells. Transient overexpression of HSP70 down-regulated the expression of orexin system in QM7 cells. Taken together, these data indicate that orexin may be a key player in stress response in avian muscle by demonstrating that heat and oxidative stress alter the expression of orexin system in quail muscle. This effect might be mediated through HSP70. Unraveling the upstream regulators and downstream effectors of orexin in avian muscle merits further in depth investigations.

## Introduction

The hypothalamic neuropeptide Orexins (ORX A and B) also known as hypocretins (HCRT1 and 2) were first identified in the lateral and posterior hypothalamus of rat brain (de Lecea et al., 1998; Sakurai et al., 1998). Both orexins are derived from a common prepro-orexin by proteolytic cleavage (Sakurai et al., 1999). The projection fields of orexin neurons were also found in numerous brain regions including the cortex, thalamus, brain stem, and spinal cord (Matsuki and Sakurai, 2008) indicating that orexin has multiple complex physiological functions. Indeed, compelling studies indicate that orexins play pleiotropic functions in mammals from appetite stimulator to sleep/wake, reward seeking, and energy homeostasis regulators (Kukkonen et al., 2002; Adamantidis and de Lecea, 2009; Carter et al., 2009; Boutrel et al., 2010).

Accumulated experimental evidence indicate that orexins and their related receptors are also expressed in peripheral tissues (Voisin et al., 2003; Heinonen et al., 2008; Okumura and Takakusaki, 2008). They signal and orchestrate their peripheral and central effects via two G protein-coupled receptors, ORXR1 and ORXR2 (Sakurai et al., 1998; Kukkonen and Leonard, 2014). Recent emerging substantiations support a role of orexin in complex stress and emotional responses. In fact, intracerebroventricular administration of orexin has been shown to activate HPA axis, increase plasma concentrations of adrenocorticotropic hormone (ACTH) and corticosterone via corticotropin-releasing hormone (CRH) receptor dependent mechanism (Jaszberenyi et al., 2000; Kuru et al., 2000; Chang et al., 2007). Initial studies have demonstrated that orexin regulates a variety of emotional, endocrine, and cardiovascular responses associated with an integrative stress response (Ciriello et al., 2003; de Oliveira and Ciriello, 2003; Kayaba et al., 2003). The most striking discovery was that orexin neurons are predominantly localized to the perifornical hypothalamus, a key panic site, and are highly responsive to panicogenic stimuli (Johnson et al., 2012a,b) indicating a key role for orexin in adaptive fight or flight responses. Orexin is also strongly present in anxiogenic brain nuclei and orexin administration has been shown to induce anxiety-like behavior probably via glutamate (Suzuki et al., 2005; Truitt et al., 2009; Henny et al., 2010; Johnson et al., 2010).

In avian (non-mammalian) species, orexin has been found also in the brain (Ohkubo et al., 2002; Miranda et al., 2013) as well as in peripheral tissues including gastrointestinal tract (Arcamone et al., 2014), pituitary gland, adrenal gland, and testis (Ohkubo et al., 2003). We recently found that orexin is expressed in avian muscle where it regulates mitochondrial bioenergetics, dynamics, and biogenesis (Lassiter et al., 2015). However, how orexin is regulated in the muscle of avian species is still unknown. This study aimed, therefore, to determine the effect of most challenging stressors (heat and oxidative stress) in poultry on orexin expression in quail muscle using *in vivo* and *in vitro* studies.

## Materials and methods

### *In vivo* experiment

The present study was conducted in accordance with the recommendations in the guide for the care and use of laboratory animals of the National Institutes of Health and the protocol was approved by the University of Arkansas Animal Care and Use Committee under protocols 13039 and 10025.

Two lines of male quails were used. These two lines were established by long-term divergent selection for circulating corticosterone response to restraint stress, after which the low stress line (resistant, R) had 66% lower plasma corticosterone levels compared to their high stress (sensitive, S) counterpart (Satterlee and Johnson, 1988). The offspring in this study was from generation 46 of the R and S quail lines. Quails of each line were hatched at the University of Arkansas Poultry Research Farm, reared separately in floor pen under environmentally controlled facilities, and were allowed *ad libitum* access to clean water and food (12.6 MJ kg^−1^, 22% protein). They were warm-brooded for 10 days at 32°C and the brooding temperature was gradually decreased each week to 24°C (thermal neutral, TN) at 4 weeks of age with a 17L:7D photoperiod cycle. At 4 weeks of age, birds of each line were exposed to two environmental conditions: acute heat stress (HS, 37°C for 1.5 h) vs. thermoneutral conditions (TN, 24°C) in a 2 × 2 factorial design. The relative humidity was 50 ± 5%. Animals were killed by cervical dislocation and leg muscle tissues were removed, immediately frozen in liquid nitrogen, and stored at −80°C for further molecular analysis.

### *In vitro* experiment

Quail muscle (QM7, Antin and Ordahl, 1991) cell lines were purchased from American Type Culture Collection (ATCC CRL-1962, Manassas, VA) and were grown in M199 medium (Life Technologies, Grand Island, NY) complemented with 10% FBS (Life Technologies), 10% tryptose phosphate (Sigma-Aldrich, St. Louis, MO), and 1% penicillin-streptomycin (Biobasic, Amherst, NY) at 37°C under a humidified atmosphere of 5% CO_2_ and 95% air. The medium was changed every 48 h and cells were subjected, during their exponential phase of growth, to the following treatments:

1. Acute heat stress exposure (HS): QM7 cells were exposed to HS (45°C) for 0.5, 1, 2, or 4 h. The control cells were maintained at 37°C.
2. Hydrogen peroxide (H_2_O_2_) treatment: Cells were treated with 10, 50, 100, or 200 μM of H_2_O_2_ (Sigma-Aldrich, St. Louis, MO) for 3 h. Untreated cells were used as control.
3. 4-Hydroxynonenal (4-HNE) treatment: Cells were treated with 10, 20, or 30 μM of 4-HNE (Sigma-Aldrich, St. Louis, MO) for 24 h. Untreated cells were used as control.
  The dose and time of the above mentioned treatments were chosen based on pilot and previous experiments (Piekarski et al., 2014).

### HSP70 plasmid preparation and transient transfection

The avian HSP70 coding region (GenBank accession No. NM_205491) was amplified by PCR using the following two oligonucleotide primers: forward 5′-AGGCACCTCCTGTTGGCGCTGCTGCT-3′ and reverse 5′-CAACTCATCCTTCTCTGCTGCTTCTT-3′. The forward primer contained the consensus Kozak sequenced fused to HSP70 coding N-terminal amino acid sequences and EcoRI restriction site. The reverse primer contained HSP70 coding C-terminal amino sequences upstream of stop codon and XbaI restriction site. The PCR product was inserted into the pGEM-T-Easy vector (Promega, Madison, WI) and further ligated into EcoRI and XbaI sites of the mammalian expression vector pcDNA3.1-V5-His vector (ThermoFisher Scientific, Waltham, MA). The orientation of the insert was confirmed by DNA sequencing.

QM7 cells were transiently transfected with pcDNA3.1-HSP70 (called hereafter pHSP70) using Lipofectamin 2000 (ThermoFisher Scientific, Waltham, MA) according to the manufacturer's instructions. Transfection was carried out for 6 h, after which the medium was removed and replaced with M199 medium containing 10%FBS and then the cells were further exposed to HS or TN condition for 4 h.

### RNA isolation, reverse transcription, and real-time quantitative PCR

Total RNAs were extracted from muscle tissues and QM7 cells by Trizol reagent (Life Technologies, Grand Island, NY) according to manufacturer's recommendations. Total RNAs were DNAse treated, reverse transcribed and amplified by QPCR as we previously described (Lassiter et al., 2015). Oligonucleotide primers specific for chicken orexin (ORX), orexin receptor 1 (ORXR1), orexin receptor 2 (ORXR2), heat shock protein 70 (HSP70), HSP60, HSP27, heat shock factor1–4 (HSF1–4), and r18S as housekeeping gene were summarized in Table 1. Relative expressions of target genes were determined by the 2^−ΔΔCt^ method (Schmittgen and Livak, 2008). For the *in vivo* and *in vitro* studies study, R quails maintained at TN conditions and control cells were used as calibrators, respectively.

**Table 1:** Oligonucleotide real-time qPCR primers.

**Gene**	**Accession number^a^**	**Primer sequence (5′ → 3′)**	**Orientation**	**Product size (bp)**
ORX	AB056748	CCAGGAGCACGCTGAGAAG	Forward	67
		CCCATCTCAGTAAAAGCTCTTTGC	Reverse	
ORXR1	NM_205505	TGCGCTACCTCTGGAAGGA	Forward	58
		GCGATCAGCGCCCATTC	Reverse	
ORXR2	AF408407	AAGTGCTGAAGCAACCATTGC	Forward	61
		AAGGCCACACTCTCCCTTCTG	Reverse	
HSP70	JO2579	GGGAGAGGGTTGGGCTAGAG	Forward	55
		TTGCCTCCTGCCCAATCA	Reverse	
HSP60	NM_001012916	CGCAGACATGCTCCGTTTG	Forward	55
		TCTGGACACCGGCCTGAT	Reverse	
HSP27	XM_001231557	TTGAAGGCTGGCTCCTGATC	Forward	58
		AAGCCATGCTCATCCATCCT	Reverse	
HSF1	L0609	GAGACGGACCCGCTGATCT	Forward	58
		GGTCGAACACATGGAAGCTGTT	Reverse	
HSF2	NM_001167764	GCCCAGCAACCAGCTTATCA	Forward	63
		TGTTCATCCAACACCAAGAAACTC	Reverse	
HSF3	L06126	CAGAGCGACGACGTCATCTG	Forward	66
		CCGCTGCTCATCCAGGAT	Reverse	
HSF4	NM_001172374	CAAAGAGGTGCTGCCCAAGT	Forward	60
		AGCTGCCGGACGAAACTG	Reverse	
18S	AF173612	TCCCCTCCCGTTACTTGGAT	Forward	60
		GCGCTCGTCGGCATGTA	Reverse	

a *Accession number refer to Genbank (NCBI)*.

*ORX, prepro-orexin; ORXR1, orexin receptor 1; ORXR2, orexin receptor 2; HSP(70, 60, 27), heat shock proteins; HSF1–4, heat shock factors*.

### Protein extraction and western blot analysis

Muscle tissues and QM7 cell homogenization, protein extraction and concentration measurement were previously described (Lassiter et al., 2015). Total proteins (70 μg) for cells and (100 μg) for tissues were assessed by immunobloting using the following polyclonal antibodies: rabbit anti-mouse ORX, rabbit anti-rat ORXR1 and 2 (Interchim, Montlucon, France), and mouse anti-HSP70 (ThermoFisher Scientific, Waltham, MA). After striping, the membrane was re-probed with GAPDH or β-actin as housekeeping proteins (Cell signaling Technology, Danvers, MA). Pre-stained molecular weight marker (precision plus protein Dual color) was used as standard (Biorad, Hercules, CA). The signal was visualized by enhanced chemiluminescence (ECL plus) (GE Healthcare Bio-Sciences, Buckinghamshire, UK) and captured by FlourChem M MultiFlour System (Proteinsimple, Santa Clara, CA). Image Acquisition and Analysis were performed by AlphaView software (Version 3.4.0, 1993–2011, Proteinsimple, Santa Clara, CA).

### Immunoflourescene

Immunoflourescene was performed as previously described (Lassiter et al., 2015) using rabbit anti-ORX, anti-ORXR1, anti-ORXR2, or mouse anti-HSP70 antibody (1:200, Interchim, Montlucon France). After incubation with Alexa Flour 488- or 594-conjugated secondary antibody (Molecular probes, Life Technologies, Grand Island, NY) and DAPI (Vector Laboratories, Burlingame, CA), images were obtained and analyzed using Zeiss Imager M2 and Axio Vision software (Carl Zeiss Microscopy, GmbH 2006–2013).

### Statistics

Data from R and S quails were analyzed by two-factor ANOVA with heat stress (HS vs. TN) and genotype (R vs. S) as classification variables. The rest of the data (oxidative stress, heat stressed cells) were analyzed by one way ANOVA. If ANOVA revealed significant effects, the means were compared by Student Newman Keuls (SNK) multiple comparison test. All data were analyzed using Graph Pad Prism software (version 6, La Jolla, CA). *P* < 0.05 was set as significantly different.

## Results

### Acute HS alters the expression of orexin system in quail muscle in a genotype-dependent manner

**Figure 1:**
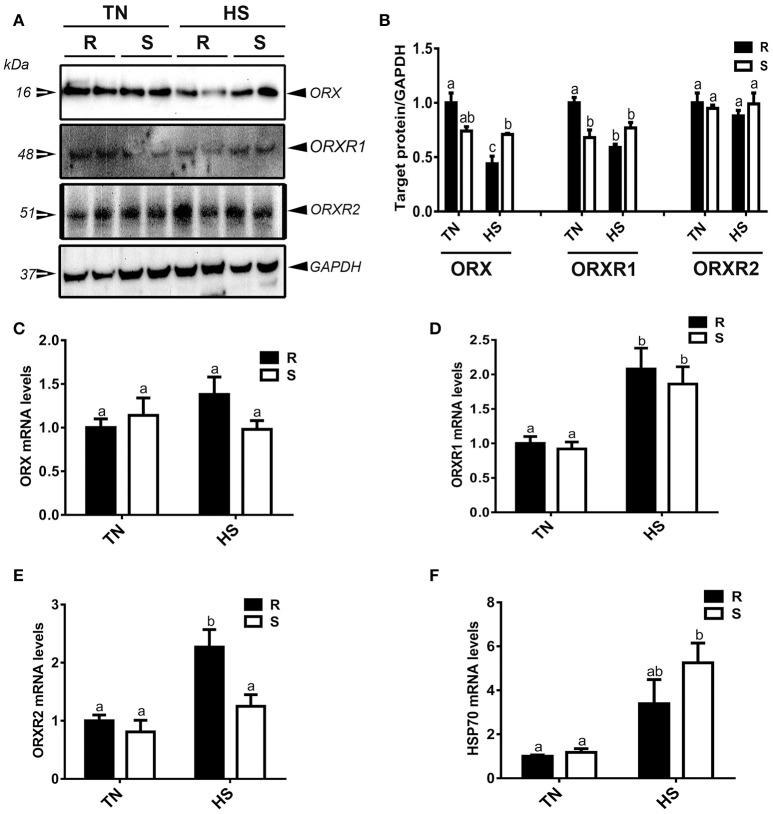
Effect of acute heat stress on the expression of orexin and its related receptors in muscle of R and S quail lines. Protein levels of ORX and orexin receptors (ORXR1/2) were determined using Western blot **(A)** and their relative expression was presented as normalized ratio of target protein/GAPDH **(B)**. Relative abundance of ORX **(C)**, ORXR1 **(D)**, ORXR2 **(E)**, and HSP70 mRNA **(F)** was measured by real-time RT-PCR. Data are presented as mean ± SEM (*n* = 6–10/group). Different letters indicate significant difference at *P* < 0.05. HS, heat stress; TN, thermoneutral; R, stress-resistant quail; S, stress-susceptible quail.

As depicted in Figure 1, acute HS significantly decreased the protein levels of both ORX and its related receptor ORXR1, but not ORXR2 (Figures 1A,B). This decrease of muscle ORX and ORXR1 expression was observed only in the R quail line resulting in a significant interaction (*P* = 0.0009 and 0.0004 for ORX and ORXR1, respectively) (Figure 1B). At the RNA level, HS significantly up regulated ORXR1 and ORXR2 but not ORX gene expression (Figures 1C–E). This significant increase of ORXR1 was observed in both quail lines, however ORXR2 mRNA abundance was more noticeable in the R quail line. HS significantly increased muscle HSP70 mRNA levels particularly in the S quail line (Figure 1F).

### Acute HS alters the expression of orexin system in QM7 cell lines

**Figure 2:**
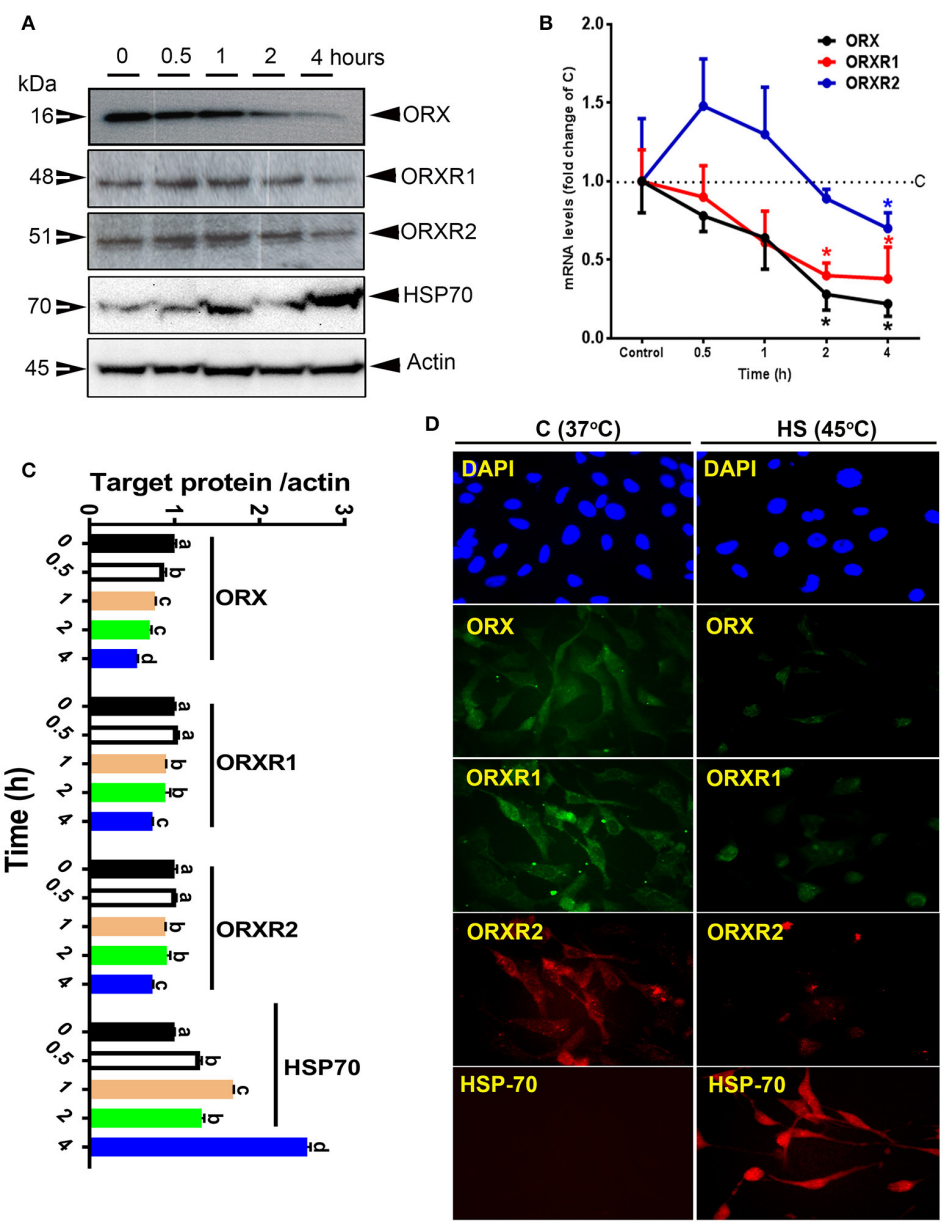
Effect of acute heat stress on the expression of HSP70 and orexin system in QM7 cell lines. Protein levels of HSP70, ORX, and ORXR1/2 were determined by Western blot **(A)** and their relative expression was presented as normalized ratio of target protein/actin **(C)**. Relative abundance of ORX and ORXR1/2 mRNA **(B)** was measured by real-time RT-PCR. Protein localization and expression was also assessed by immunofluorescence **(D)**. Data are presented as mean ± SEM (representative of at least three replicates). ^*^ and different letters indicate significant difference at *P* < 0.05.

Acute HS significantly down-regulated the expression (mRNA and protein levels) of ORX and its related receptors ORXR1 and ORXR2 in QM7 cell lines (Figures 2A–C). This down-regulation was time-dependent; it started at 30 min and reached significant levels at 4 h post-treatment for both mRNA abundance and protein levels of ORX, ORXR1, and ORXR2 (Figures 2A–C). HS induced HSP70 expression (Figure 2C). These changes were also confirmed by immunofluorescence staining (Figure 2D).

### HSP70 overexpression alters the expression of orexin system in QM7 cells

**Figure 3:**
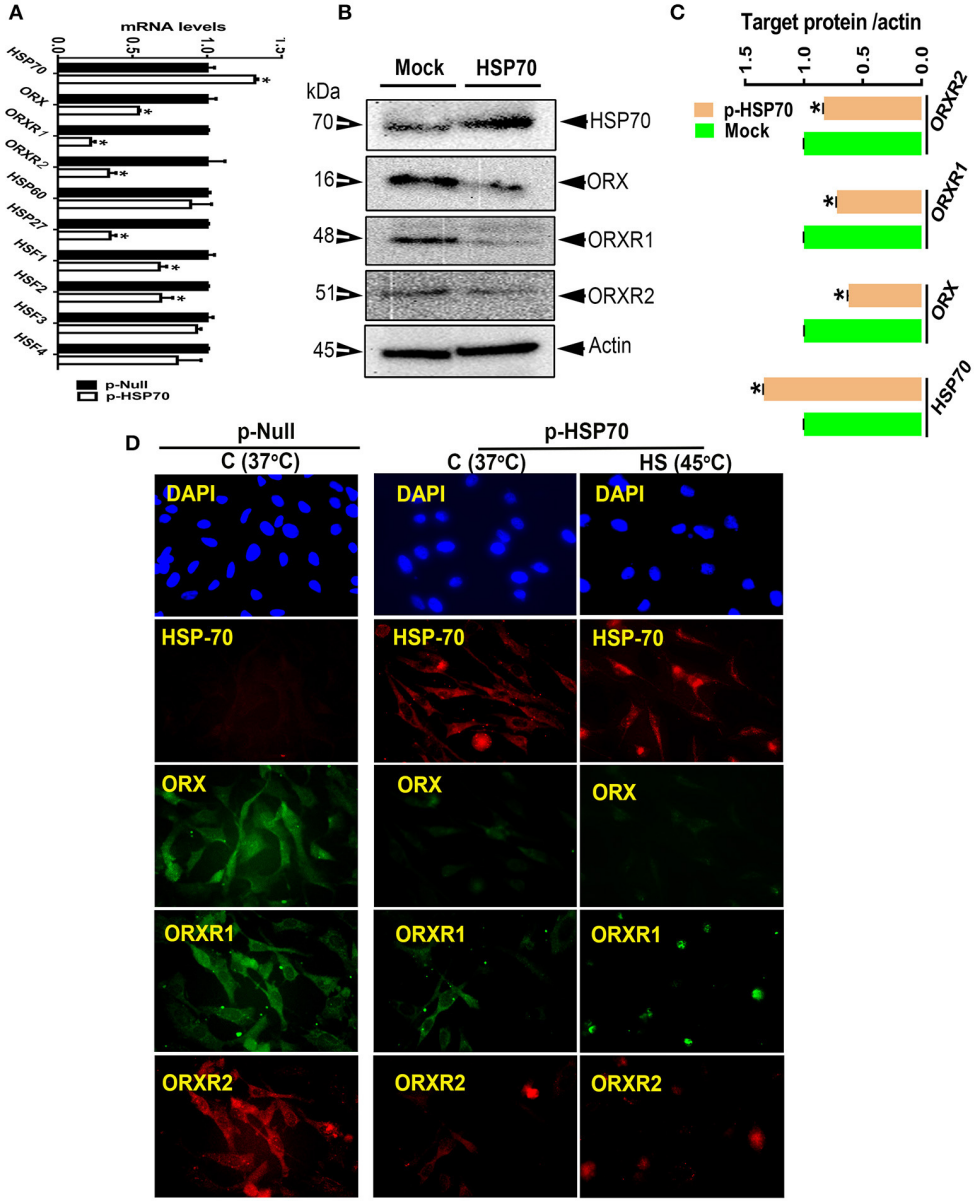
Effect of HSP70 overexpression on the expression of orexin and its related receptors in QM7 cells. Overexpression of HSP70 down-regulates the expression of ORX and ORXR1/2 at both mRNA and protein levels measured by real-time RT-PCR **(A)**, Western blot **(B,C)**, and immunofluorescence **(D)**. Data are presented as mean ± SEM (representative of at least three replicates). ^*^ indicate significant difference at *P* < 0.05.

Overexpression of HSP70 gene by 32% results in 34% increase in HSP70 protein levels and a significant decrease in both mRNA and protein abundance of ORX (46 and 38% for mRNA and protein levels, respectively), ORXR1 (78 and 28% for mRNA and protein levels, respectively), and ORXR2 (66 and 17% for mRNA and protein levels, respectively) (Figures 3A–C). HSP70 upregulation reduces the expression of HSP27 by 65% (*P* < 0.05), HSF1 by 32% (*P* < 0.05), and HSF2 by 31% (*P* < 0.05) but not that of HSP60, HSF3, or HSF4 (Figure 3A). Exposure of HSP70-transfected QM7 cells to HS for 4 h increased further the expression of HSP70 and decreased that of ORX and its related receptors (ORXR1 and ORXR2) compared to cells (control or HSP-70 transfected) maintained at 37°C (Figure 3D).

### Oxidative stress alters the expression of orexin system in QM7 cells

**Figure 4:**
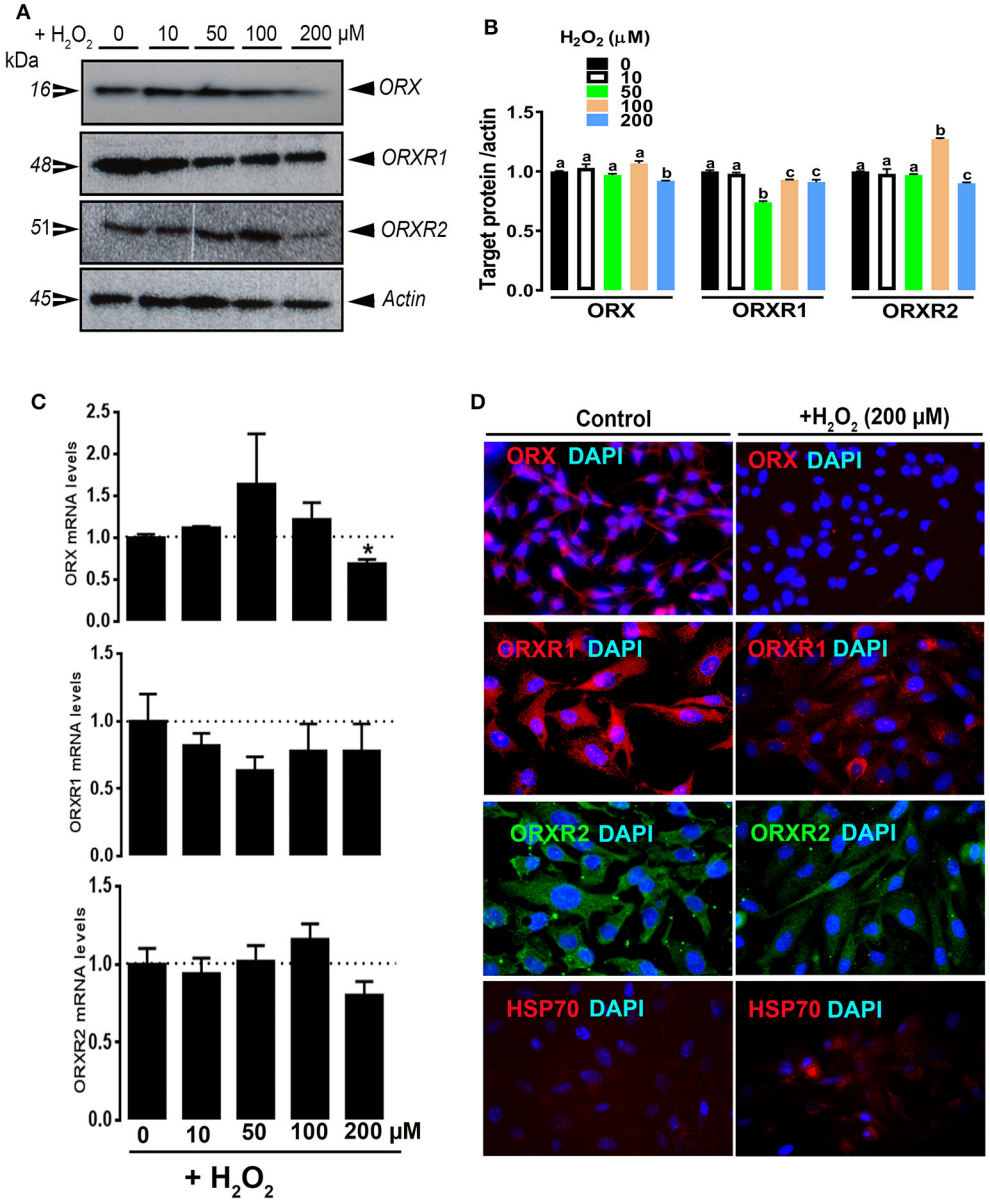
Effect of H_2_O_2_ on ORX and ORXR1/2 expression in QM7 cells. QM7 Cells were treated with 10, 50, 100, and 200 μM of H_2_O_2_ for 3 h. Untreated cells were used as control. ORX and ORXR1/2 protein levels were determined by Western blot **(A,B)** and immunofluorescence **(D)**. The relative expression of target proteins was presented as normalized ratio of target protein/actin **(B)**. ORX and ORXR1/2 mRNA abundances were measured by qPCR using 2^−ΔΔCt^ method **(C)**. Data are presented as mean ± SEM (representative of at least three replicates). ^*^ and different letters indicates a significant difference between H_2_O_2_-treated and untreated cells (*P* < 0.05).

**Figure 5:**
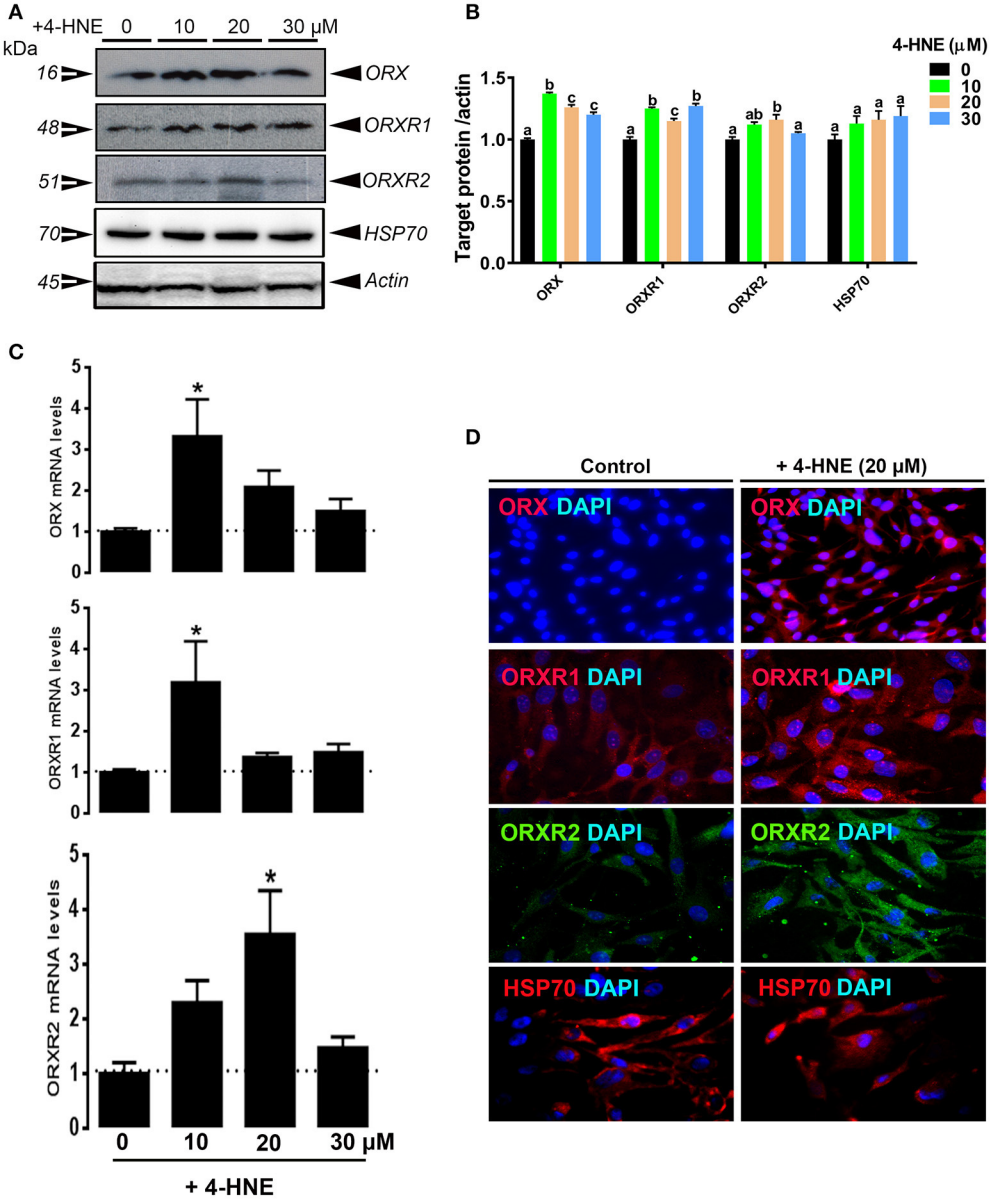
Effect of 4-HNE on ORX and ORXR1/2 expression in QM7 cells. QM7 Cells were treated with 10, 20 and 30 μM of 4-HNE for 24 h. Untreated cells were used as control. ORX and ORXR1/2 protein levels were determined by Western blot **(A,B)** and immunofluorescence **(D)**. The relative expression of target proteins was presented as normalized ratio of target protein/actin **(B)**. ORX and ORXR1/2 mRNA abundances were measured by qPCR using 2^−ΔΔCt^ method **(C)**. Data are presented as mean ± SEM (representative of at least three replicates). ^*^ and different letters indicates a significant difference between 4-HNE-treated and untreated cells (*P* < 0.05).

Treatment of QM7 cells with high dose (200 μM) of H_2_O_2_ significantly induced HSP70 expression (Figure 4D) and significantly decreased the protein levels of ORX, ORXR1, and ORXR2 (Figures 4A,B,D). ORX mRNA abundances were also significantly decreased by H_2_O_2_, however ORXR1 and ORXR2 gene expression remained unchanged (Figure 4C). Administration of 4-HNE did not elicit any change on HSP70 expression (Figures 5A,D). However, it had a biphasic effects on the gene expression of orexin system with an upregulation at low doses (10 and 20 μM) and down-regulation at higher dose (30 μM) (Figure 5C). At protein levels, all administrated doses of 4-HNE significantly increased ORX and ORXR1 expression (Figures 5A,B). ORXR2 protein levels, however, were upregulated only by the 20 μM of 4-HNE and not by the other doses (Figures 5A,B).

## Discussion

The objective of the present study was to determine the effects of acute heat load and oxidative stress, most challenging stressors to poultry production, on the expression of orexin system in quail muscle. Large, abrupt, and widespread extreme heat waves have occurred repeatedly in the past (Alley et al., 2005) and are predicted to increase for the next century (Mora et al., 2013). High ambient temperature can be devastating to commercial poultry production from its adverse effects on energy intake depression, productive efficiency suppression, well-being alteration, and mortality (Dale and Fuller, 1980; Cahaner and Leenstra, 1992; Leenstra and Cahaner, 1992; Deeb and Cahaner, 2002; Deeb et al., 2002). This, in turn, results in estimated total annual economic loss to the poultry industry of more than 150 million dollars (St-Pierre et al., 2003) and such harm will be higher during the next decade as more frequent and intense heat waves are projected (Mora et al., 2017). There is, therefore, a critical need for mechanistic understanding of heat stress responses in avian species at cellular and molecular levels that may help for a subsequent development of innovative strategies (nutrition, genetic selection, and/or management) to alleviate heat stress and improve bird thermo-tolerance.

The expression of orexin system in the avian muscle and its alteration by heat stress in the current study indicates that orexin may play a key role in heat stress response. Orexin has been extensively reported to have multiple physiological functions not only in feeding and energy metabolism but also in wakefulness and reward in mammals. Accumulating evidence supports also an integrative role for orexin in nociceptive perception, pain regulation, and stress-related behaviors in rodents. Intrathecal administration of orexin peptide has been shown to reduce nociceptive responses in a mouse model of thermal, inflammatory, and visceral pain (Yamamoto et al., 2002). Recently, Inutsuka and co-workers elegantly demonstrated that adult-stage selective ablation of orexin neurons enhances pain-related behaviors, while activation of orexin neurons induces analgesia (Inutsuka et al., 2016). Similarly, Bingham et al. reported that intracerebroventricular injection of orexin produces an analgesic effect in the rat hot plate test (Bingham et al., 2001). Despite the species-specific differences in orexin system, our present data in combination with previous rodent studies suggest that orexin might be involved in thermal nociceptive transmission and heat stress response in avian species. However, the mechanism and the role (pro- or anti-analgesic/pain) of orexin in avian muscle under heat stress challenges remain unclear at this time-point and further in depth investigations are warranted.

In contrast to our previous study where we have shown that heat stress down-regulated the expression of orexin at both mRNA and protein levels in quail liver (Greene et al., 2016), heat stress dysregulated only orexin protein but not mRNA abundances in quail muscle in the current study. This indicated that heat stress might regulate orexin expression at post-transcriptional and/or translational levels in quail muscle (Sakurai et al., 1998; Cai et al., 2000; Chen and Randeva, 2010). The similar expression pattern observed here between orexin and ORXR1 proteins in the muscle of heat-stressed quails indicates that ORXR1, but not ORXR2, might be more sensitive and responsive to orexin dysregulation-induced by heat stress. Like orexin, the regulation of ORXR1 and ORXR2 expression by heat stress seems to occur at post-transcriptional/translational levels since the mRNA abundances were up regulated for both receptors, while the protein levels were decreased or unchanged for ORXR1 and ORXR2, respectively (Chen and Randeva, 2010). The absence of correlation between gene and protein expression of orexin system in our study indicates that their transcription and translation are differentially regulated by HS. It is possible that under our experimental stress conditions, the pool of already synthesized ORX/ORXR1 mRNA might not be efficiently translated with a slow turnover or degradation, however its protein product accumulation decreases. The protein stability may also decrease by post-translational modification like acetylation or glycosylation (Thompson et al., 2014). Interestingly, the down-regulation of orexin and ORXR1 expression is more pronounced in the muscle of R compared to S quail lines indicating that the regulation of muscle orexin system by heat stress is genotype-dependent. Such results have been previously observed on the effect of orexin on both hippocampal clock- and circadian oscillation-related genes in APP/PS1dE9 vs. wild-type mice (Ma et al., 2016). Furthermore, a genotype-dependent differences in sleep and vigilance, which is controlled by orexin system, has been reported (Landolt, 2008).

As HSP70 expression is induced by HS and as HSP70 is now understood to regulate gene transcription and cellular signaling, we questioned whether the orexin dysregulation-induced by heat stress might be mediated by HSP70 using *in vitro* (QM7 cells) system. As for the *in vivo* study, we first showed that HS induced HSP70 expression and down-regulated ORX mRNA and protein levels in a time-dependent manner in QM7 cells which indicates that HS may directly alter the expression of orexin system. We next transiently overexpressed HSP70 in QM7 cells and found reduced protein levels of orexin and its related receptors which is further dysregulated by HS. Together, these data suggest that HS may alter the expression of orexin system in quail muscle via HSP70. The role of heat shock proteins in gene-transcriptional regulation is only at the beginning of being explored. As proof of concept, Jin and co-workers have recently shown that knockdown of HSP70 led to reduction of matrix metalloproteinase 9 (MMP9) mRNA expression and WASF3/Wave3 protein levels in bladder cancer (BC) cell line (Jin et al., 2017). Moreover, Liang et al. demonstrated that Runx2 gene was transcriptionally regulated by HSP90 via the AKT/GSK-3β/β-catenin signaling pathway in human OS Saos-2 cell lines (Liang et al., 2017). Although it is still unclear how HSP70 regulate muscle orexin expression in heat-stressed quails, it is possible though that HSF1-altering RNA polymerase II might be involved (Mahat et al., 2016). As an additional potential mechanism, HSP70 might result in destabilization of orexin protein through proteasome degradation (Teng et al., 2012).

As heat load has been reported to induce intracellular oxidative stress including formation of H_2_O_2_ and 4-HNE (Cheng et al., 2001; Bruskov et al., 2002), we sought to determine the effect of H_2_O_2_ and 4-HNE on orexin system expression in QM7 cells. Similar to heat stress, H_2_O_2_ treatment dysregulated the expression of orexin system in QM7 cells suggesting that the effect of heat stress might be mediated through induction of oxidative stress and formation of reactive oxygen species (ROS) which merits further in depth investigations. Hepatic orexin expression has been previously shown by our group to be altered by H_2_O_2_ (Greene et al., 2016) and H_2_O_2_/ROS has recently become a crucial player in regulating neuronal response in a substrate-dependent manner (Kuo et al., 2011; Drougard et al., 2015). As H_2_O_2_/ROS are now known to act in many signaling pathways in different peripheral organs, the induction of phosphorylated AMPKα1/2 at Thr172 site in the current study (data not shown) suggest that heat and oxidative stress might regulate muscle orexin expression via AMPK pathway. In fact, as for heat stress, ROS has been reported to reduce mitochondrial ATP synthesis, leading to an increased AMP/ATP ratio and subsequent phosphorylation of AMPKα1/2-Thr^172^ by LKB1 and CaMKKβ (Morales-Alamo and Calbet, 2016). Recently, AMPK has also been shown to be involved in the regulation of orexin expression in mammals (Martins et al., 2016). It is also possible that orexin might be susceptible to mis-folding under higher doses of oxidative stress (H_2_O_2_ and 4-HNE) and thereby is subject to ubiquitination and proteasome-mediated degradation (Zhan et al., 2011).

In conclusion, the findings of the current study are the first evidence of orexin system regulation by heat and oxidative stress in quail muscle and unveil a potential key role of HSP70 in controlling orexin expression. Further studies investigating the up- and down-stream cascades involved in the regulation and effects of orexin on avian muscle biology are warranted.

## Author contributions

SD: conceived and designed the study; PN and EG: conducted the animal care, sample collection, and data analyses; SD: wrote the paper with a critical review by all authors (NA, WB, PN, B-WK, and EG).

### Conflict of interest statement

The authors declare that the research was conducted in the absence of any commercial or financial relationships that could be construed as a potential conflict of interest.
